# Polymer Composites in 3D/4D Printing: Materials, Advances, and Prospects

**DOI:** 10.3390/molecules29020319

**Published:** 2024-01-09

**Authors:** Ayyaz Mahmood, Fouzia Perveen, Shenggui Chen, Tayyaba Akram, Ahmad Irfan

**Affiliations:** 1 School of Mechanical Engineering, Dongguan University of Technology, Dongguan 523808, China; ayyazcmc@gmail.com; 2 School of Life Science and Technology, University of Electronic Science and Technology, Chengdu 610054, China; 3 School of Art and Design, Guangzhou Panyu Polytechnic, Guangzhou 511483, China; 4 Dongguan Institute of Science and Technology Innovation, Dongguan University of Technology, Dongguan 523808, China; 5 School of Interdisciplinary Engineering & Sciences (SINES), National University of Sciences and Technology (NUST), Sector H-12, Islamabad 44000, Pakistan; 6 Department of Physics, COMSATS Institute of Information Technology, Lahore 54000, Pakistan; 7 Department of Chemistry, College of Science, King Khalid University, P.O. Box 9004, Abha 61413, Saudi Arabia

**Keywords:** additive manufacturing, 3D printing, 4D printing, polymers, shape memory

## Abstract

Additive manufacturing (AM), commonly referred to as 3D printing, has revolutionized the manufacturing landscape by enabling the intricate layer-by-layer construction of three-dimensional objects. In contrast to traditional methods relying on molds and tools, AM provides the flexibility to fabricate diverse components directly from digital models without the need for physical alterations to machinery. Four-dimensional printing is a revolutionary extension of 3D printing that introduces the dimension of time, enabling dynamic transformations in printed structures over predetermined periods. This comprehensive review focuses on polymeric materials in 3D printing, exploring their versatile processing capabilities, environmental adaptability, and applications across thermoplastics, thermosetting materials, elastomers, polymer composites, shape memory polymers (SMPs), including liquid crystal elastomer (LCE), and self-healing polymers for 4D printing. This review also examines recent advancements in microvascular and encapsulation self-healing mechanisms, explores the potential of supramolecular polymers, and highlights the latest progress in hybrid printing using polymer–metal and polymer–ceramic composites. Finally, this paper offers insights into potential challenges faced in the additive manufacturing of polymer composites and suggests avenues for future research in this dynamic and rapidly evolving field.

## 1. Introduction

The world of manufacturing is undergoing a profound and transformative evolution, and at the heart of this revolution lies the ground-breaking technology of additive manufacturing, more commonly known as 3D/4D printing [1,2,3,4,5]. This revolutionary approach allows for the meticulous layer-by-layer deposition of materials to create intricate and bespoke objects, thereby altering the very foundations of how we conceive, design, and manufacture [6,7,8,9,10,11,12]. Since its inception and subsequent commercialization, 3D printing has witnessed widespread adoption across a spectrum of industries, notably engineering, manufacturing, healthcare, aerospace, and the automotive sector. Its applications, ranging from rapid prototyping to the fabrication of intricate lightweight structures, have brought about a transformative wave of innovation in contemporary manufacturing. The impact of 3D printing on the landscape of industrial part/component design and equipment development is profound [1,2,3,4,13,14]. This revolutionary technology has not only challenged but also transcended the limitations of conventional manufacturing methods. It has empowered manufacturers and researchers to embark on endeavors that were once deemed unattainable using traditional processes. Over the past three decades, the relentless pursuit of enhancements and innovations has fueled the remarkable evolution of 3D printing technology [5,11,15,16,17,18,19].

**Figure 1:**
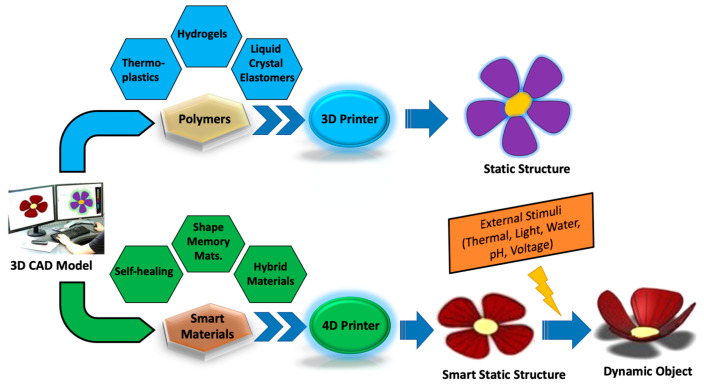
Schematics of the overall 3D and 4D printing process using polymeric materials.

Central to this innovative transformation are polymeric materials, a remarkably diverse class of substances that offer unmatched versatility, accessibility, and opportunities in the realm of additive manufacturing. Polymeric materials, also commonly referred to as plastics, have gained substantial traction within the domain of additive manufacturing [10,20]. Their appeal stems from their exceptional adaptability and widespread availability [21,22]. These materials are the cornerstone of an ever-expanding range of applications, enabling the production of prototypes, intricate components, and customized solutions across a spectrum of sectors, including aerospace, healthcare, and consumer goods. This transformative shift in the additive manufacturing landscape is significantly facilitated by the intrinsic advantages that polymeric materials bring to the table. Schematics of the overall 3D and 4D printing process are shown in Figure 1.

Foremost among these advantages is the acceleration of product development cycles. Additive manufacturing, in conjunction with polymeric materials, empowers engineers and designers to seamlessly transition digital designs into tangible prototypes, facilitating rapid testing, validation, and redesign processes. This, in turn, results in remarkable time and resource savings, bolstering the speed and efficiency of product development [5,11,12,13,15,16,17,18,19]. Moreover, additive manufacturing offers a fresh perspective on the design and geometry of creations. It allows the realization of complex and intricately structured components that were previously unattainable using traditional manufacturing techniques. This capability to reimagine and innovate geometrically enables designs that are not only aesthetically appealing but also functionally superior. In addition to these transformative advantages, additive manufacturing contributes significantly to sustainability initiatives [7,18,23]. It aids in minimizing material wastage, reducing energy consumption, and minimizing the carbon footprint of various industries. By its very nature, additive manufacturing is a process that generates minimal waste and, through localized production, reduces the need for long-distance transportation. This is a crucial step forward in reducing carbon emissions and aligning the manufacturing industry with the noble objectives of a more sustainable future.

This comprehensive review navigates the intricate landscape of additive manufacturing with a focus on polymeric materials, providing an in-depth exploration of recent advancements, challenges, and future trajectories in this transformative technology. Our goal is to offer a holistic perspective on how polymeric materials within the realm of additive manufacturing are reshaping the realms of creativity, efficiency, and sustainability in diverse domains.

### The Evolution of Polymer 3D Printing: A Journey through Time

**Figure 2:**
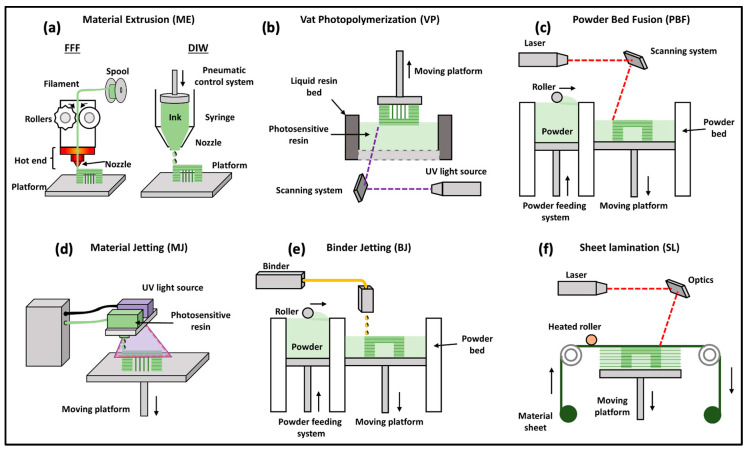
Illustration of the historical progression of 3D printing and the advancements in polymer materials for 3D printing, marking a shift from rapid prototyping to scalable and tailor-made production. Figure reproduced with permission from [21].

The history of polymer 3D printing is a fascinating tale of innovation and technological progress. This narrative unfolds alongside the timeline of 3D printing, with polymer materials playing a pivotal role. The development of feedstock materials, especially polymers, has been instrumental in shaping the world of 3D printing as we know it today. The chronological history of 3D printing, along with the evolution of polymer materials, is depicted in Figure 2. It is important to note that the foundations for many of the polymers used in 3D printing today, such as polyamides, polylactic acid (PLA), and epoxy, were laid during the 1920s and 1940s [20]. These early discoveries set the stage for the polymer-based 3D printing revolution. The conceptual groundwork for additive manufacturing (AM) was laid over 50 years ago, highlighting the longstanding fascination with the idea of creating objects layer by layer. However, it was not until the 1980s and 1990s that we witnessed the birth and rapid growth of 3D printing as a practical technology. The watershed moment came in 1987 when stereolithography, one of the first 3D printing methods, was commercialized. This was a significant leap forward, and it marked the beginning of a new era.

The 1980s and 1990s also saw the invention of other ground-breaking 3D printing techniques, including fused filament fabrication (FFF) and selective laser sintering (SLS) [24]. These methods, which are still widely used today, further expanded the horizons of 3D printing technology. The late 1990s brought a surge of innovation as various versions and iterations of 3D printing techniques emerged, thanks to the concurrent advancements in computer technologies. This period of rapid development paved the way for the widespread exploration of 3D printing applications in various industries. In the early 2000s, 3D printing started to find its footing in industries like medicine and aerospace, where its potential for creating complex and customized parts was particularly valuable. Simultaneously, many early patents for 3D printing techniques began to expire, allowing numerous companies to enter the 3D printing market. This accessibility brought 3D printers within reach of the public, democratizing a technology that was once confined to the realm of research and development. The journey of polymer 3D printing is a testament to human ingenuity and the relentless pursuit of innovation. As technology continues to advance, the future of polymer 3D printing holds the promise of even more remarkable developments and widespread applications.

In the last decade, 3D printing technology has undergone a transformative journey, marked by significant advancements that have greatly elevated its precision, accuracy, and speed. The initial stages of additive manufacturing predominantly involved the adaptation of existing polymer materials to suit the demands of 3D printing. However, the landscape has rapidly evolved, and today, there is a growing emphasis on the development of polymers uniquely tailored for 3D printing. This progress has been accelerated by the rapid emergence of high-performance 3D printing materials. These cutting-edge materials encompass a broad spectrum, including intelligent polymers and advanced engineering plastics. The synergistic integration of these materials with additive manufacturing has catalyzed a new industrial revolution across various sectors. A notable example of these pioneering materials is the category of shape memory polymers [25,26,27,28], which exhibit the remarkable ability to “remember” and return to their original shape under certain conditions. Smart hydrogels [29,30,31], another breakthrough, demonstrate sensitivity to environmental stimuli, enabling them to change volume or shape in response to factors like temperature, moisture, or pH levels. The advent of liquid crystal polymers (LCPs) and liquid crystal elastomers (LCEs) [32,33,34,35] further exemplifies this paradigm shift. These materials, characterized by their anisotropic properties and molecular orientation control in response to temperature fluctuations, have ushered in a new era typified by 4D printing. The concept of 4D printing encompasses the creation of objects that can undergo dynamic transformations over time in response to external triggers, representing a paradigm shift in the realm of manufacturing. This innovative approach has unlocked new avenues for the production of smart devices, advanced robotics, and cutting-edge biomedical products [18,19,36,37,38]. The fusion of novel materials with 3D printing technology has ushered in a new era of limitless possibilities, giving industries the tools to fabricate smart, adaptive, and innovative solutions that were once considered beyond reach.

## 2. Polymer Materials and Design for 3D Printing

Three-dimensional printing technology is fundamentally driven by digital models, where physical objects are created by adding successive layers of printing materials. Various methods are used for the preparation of polymer composites, each with its unique advantages and disadvantages. These methods include deposition molding, selective laser sintering, ink-jet 3D printing, stereolithography, and 3D drawing, among others. While some technologies are well-established and widely adopted, others are still in the research and development stage or are used by a limited number of scientists. The choice of a specific 3D printing technology depends on several factors, such as the raw material requirements, processing speed and precision, cost considerations, and the final performance characteristics of the products to be manufactured. Each method has its own set of strengths and limitations, making it crucial to match the chosen technology with the specific requirements of the intended application [22]. These methods are briefly discussed as follows. Deposition molding: This method involves depositing successive layers of materials, often in filament or granular form, to build the final object. It is widely used and suitable for a variety of applications due to its relatively simple and cost-effective nature. Selective laser sintering (SLS): In this approach, a laser is used to selectively sinter powdered material, typically thermoplastic polymers or metals, to create the desired object. It is renowned for producing high-quality, functional parts with good mechanical properties. Ink-jet 3D printing: This method operates on the principle of depositing liquid material (often polymers) in a layer-by-layer fashion using an inkjet-style print head. It is valued for its ability to create full-color and multi-material prints, making it popular in applications requiring vibrant aesthetics. Stereolithography (SLA): SLA is a photopolymerization process that utilizes a UV laser to solidify liquid resin layer by layer. Additionally, some SLA systems use a DLP projector to display the UV image of each layer, allowing simultaneous exposure of the entire layer. The advantage of using a DLP projector lies in its ability to expose an entire layer simultaneously, potentially speeding up the printing process compared with systems that use a laser to trace each layer point by point. SLA is renowned for its high resolution and precision, making it suitable for creating detailed and intricate objects. Three-dimensional drawing: This method typically involves manually controlling a 3D printing pen, which extrudes a thermoplastic filament to create freeform structures. It is a versatile and accessible option for artists, designers, and hobbyists. Further extensive details of these printing methods can be found in the literature [4,6,12,39,40,41,42,43].

**Figure 3:**
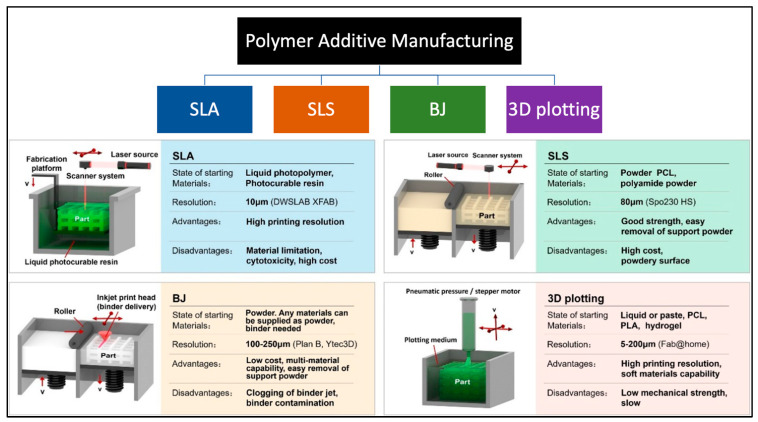
The evolving landscape of polymer 3D printing technologies for varied applications. Figure reproduced with permission from [12].

The choice of polymer 3D printing technology should be made after a thorough assessment of the unique requirements and constraints of the intended application. While some technologies may excel in precision and detail, others may prioritize speed and cost-effectiveness. Additionally, as the field of 3D printing continues to evolve, it is essential to stay informed about emerging technologies that may offer even more advanced capabilities in the near future. The dynamic landscape of polymer 3D printing technologies is illustrated in Figure 3, showcasing the options available and their respective applications.

### 2.1. Thermoplastics

**Figure 4:**
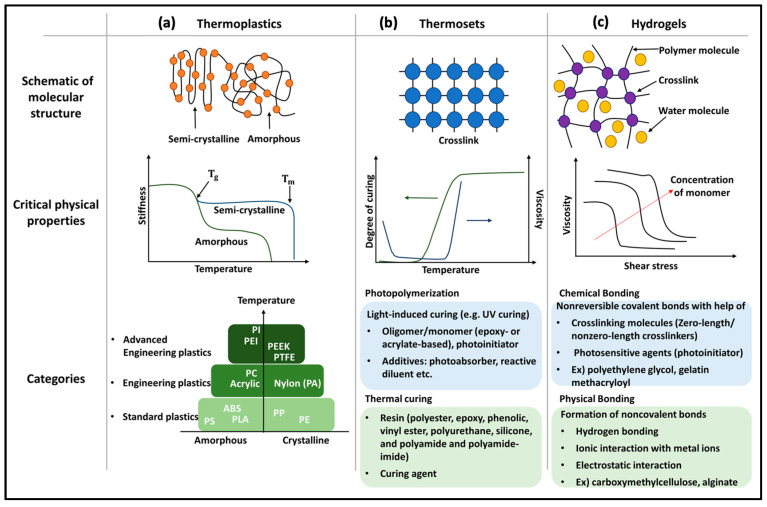
Frequently utilized polymers in 3D printing along with their respective properties: (**a**) thermoplastics, (**b**) thermosets, and (**c**) hydrogels.

Thermoplastic polymers, commonly utilized for their ability to soften when heated and solidify upon cooling, exhibit distinctive characteristics based on their molecular structure, as depicted in Figure 4a. Amorphous thermoplastics possess a randomly ordered molecular structure without a distinct melting point, facilitating thermoforming. In contrast, semi-crystalline thermoplastics feature a highly organized molecular structure with a well-defined melting point, transitioning abruptly from solid to liquid upon heat absorption. The strong intermolecular forces in semi-crystalline polymers contribute to their favorable mechanical properties. Notably, all thermoplastic polymers offer reversible processing capabilities, rendering them well-suited for extrusion-based 3D printing.

**Figure 5:**
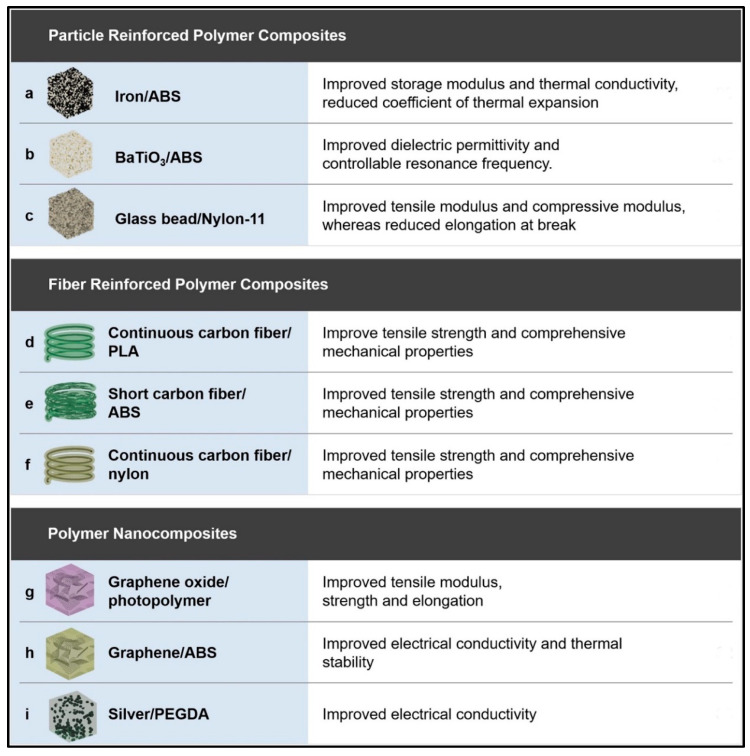
Polymer composite reinforcement methods and examples. Classification and instances of reinforcement techniques utilized in polymer composites: (**a**) iron/acrylonitrile butadiene styrene (ABS) composite [46], (**b**) BaTiO_3_/ABS composite [47], (**c**) glass bead/nylon-11 composite [48], (**d**) continuous carbon fiber/polylactic acid (PLA) composite [49], (**e**) short carbon fiber/ABS composite [50], (**f**) continuous carbon fiber/nylon composite [51], (**g**) graphene oxide/photopolymer composite [52], (**h**) graphene/ABS composite [53], and (**i**) silver/poly(ethylene glycol) diacrylate (PEGDA) composite [54]. Figure reproduced with permission from [12].

The mechanical characteristics of pure thermoplastic materials in their pristine state might not be adequate for specific applications, necessitating modifications to bolster their properties for fused filament fabrication (FFF). Ning et al. [44] embarked on a study where they combined plastic particles, specifically acrylonitrile butadiene styrene (ABS), with carbon fiber during the FFF process. This incorporation of 5 wt% carbon fiber led to notable enhancements in bending stresses, bending moduli, and bending toughness compared with pure plastic, marking increases of 11.82%, 16.82%, and 21.86%, respectively. It is noteworthy that the samples containing 10% carbon fiber displayed the highest porosities, as indicated in Figure 5. Another investigation conducted by Tekinalp et al. [45] revolved around the utilization of short carbon fibers (ranging from 0.2 to 0.4 mm) as an additive to ABS for 3D printing using FFF technology. This approach led to remarkable increases in tensile strength and the modulus of elasticity of the 3D-printed samples, showing improvements of around 115% and 700%, respectively, in comparison with traditionally molded composites. Despite the relatively high porosities observed in 3D-printed composites, they consistently exhibited substantial tensile strengths and moduli.

Tian et al. [49] introduced long carbon fibers as a reinforcement to a polylactic acid (PLA) matrix, creating a raw silk material suitable for 3D printing using the FFF process. By fine-tuning the process parameters, they managed to achieve impressive results. The 3D-printed samples, containing 27% long carbon fibers, exhibited maximum bending tensile strengths and elastic moduli reaching 335 MPa and 30 GPa, respectively. These enhancements in mechanical properties rendered the printed samples potentially useful in aerospace applications. Furthermore, Eutionnat-Diffo et al. [55], using FFF, delved into the relationship between the tensile deformation of non-conductive and conductive PLA filaments deposited on polyethylene terephthalate (PET) fabrics, the fabric properties, and the printing platform temperature. Their research also involved the presentation of a theoretical and statistical optimization model. Notably, they observed that the non-conductive PLA printing guide demonstrated improved durability after washing or with the addition of a conductive filler. However, the washing process had an impact on the fracture stress of the woven fabric after printing.

**Figure 6:**
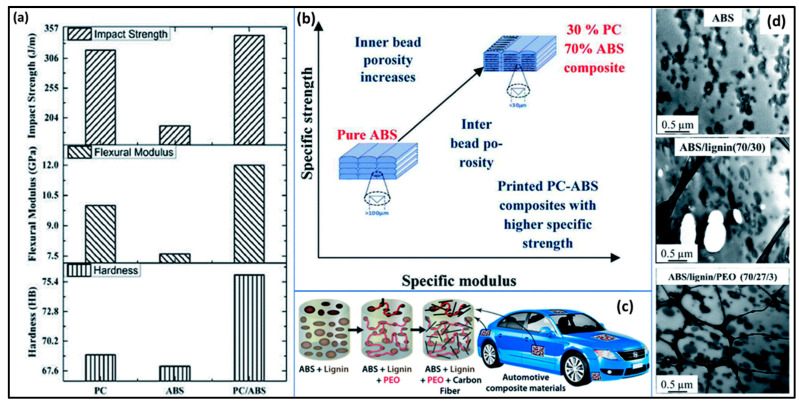
(**a**) A comparison of the mechanical properties of polycarbonate (PC), ABS, and PC/ABS-based specimens. (**b**) Schematic representation illustrating the difference in strength and modulus for 3D-printed pure ABS and its composites using the FFF technique. Reproduced with permission from [68]. (**c**) Schematic representation showcasing the formulation of ABS composites using lignin, poly(ethylene oxide) (PEO), and carbon fibers for automotive applications. (**d**) Transmission electron microscopy images of neat ABS, ABS/30% lignin, and ABS/27% lignin/3% PEO blends. Reproduced with permission from [70].

ABS exhibits compatibility with various materials, including styrene ethylene butadiene styrene, ultrahigh-molecular-weight polyethylene [56], montmorillonite clay [57], layered silicates [58], and other petroleum-based and biobased substances. This article focuses on the recent advancements in 3D printing utilizing ABS, a versatile polymer widely applicable in automotive applications, electrical devices [59,60], custom-fit prosthetics [61], aerospace, energy storage [62,63], artifact complex designs [64], multi-purpose sensor designs [65,66], and dielectric devices [67]. ABS was reinforced with polycarbonate (PC) in different proportions (10, 20, and 30 wt%), and the resulting ABS/PC composite filaments were used in FFF-based 3D printing [68]. The mechanical properties of 3D-printed pure ABS, pure PC, and ABS/PC composite samples were assessed and compared, revealing enhanced flexural modulus, tensile strength, and flexural strength in ABS/PC composites due to modified void formation and improved interfacial bonding (Figure 6a,b). Chen et al. [69] conducted an analysis of the scratch resistance and mechanical performance of 3D-printed ABS specimens, both before and after the addition of poly(methyl methacrylate) (PMMA). The incorporation of 30% PMMA resulted in a significant improvement in scratch resistance, hardness, melt flow rate, mechanical performance, and surface glossiness of ABS. Additionally, the toughness of ABS/PMMA blends was enhanced by introducing small quantities of methacrylate–butadiene–styrene (MBS) as a toughness modifier.

In a another study, the FFF-based 3D printing of ABS with biomass-derived lignin (10, 20, and 30 wt%) was investigated, following compatibilization with 10 wt% poly(ethylene oxide) (PEO) [70], as depicted in the schematic representation in Figure 6c. The findings indicated that a lower percentage of lignin in ABS led to a decrease in tensile strength, which was partially mitigated by the inclusion of PEO in ABS/lignin composites, reducing the activation energy of the blend via improved plasticization of the hard phase. The dispersion and dimensions (300–1000 nm) of lignin in ABS significantly contributed to the enhanced mechanical properties. With PEO as an interfacial adhesion promoter, the dimensions of lignin particles were further reduced to the range of 200–500 nm (as shown in Figure 6d). Additionally, the incorporation of chopped carbon fibers (20 vol%) into the ABS/lignin/PEO composite system resulted in 3D-printed samples with superior mechanical strength (77–80 MPa), demonstrating its potential application in the fabrication of automotive parts.

Another study by Shim et al. [71] investigated the influence of varying printing orientations (0°, 45°, and 90°) when using PMMA for 3D printing of denture base resin. Their comprehensive study revealed that the selected printing orientation played a pivotal role in determining key factors such as print resolution, bending strength, surface morphology, and microbial reactions. Consequently, their research highlights the significance of precise printing direction selection for achieving superior performance in denture resin 3D printing.

### 2.2. Thermosetting Polymers

In contrast to thermoplastic polymers that soften when heated and harden when cooled, thermosetting polymers undergo a chemical transformation during the initial heating process that sets them apart. This transformation results in the formation of cross-linked structures within the polymer matrix, making them rigid and stable upon cooling. Crucially, in contrast to thermoplastics, thermosetting polymers cannot be melted or reshaped when subjected to heat again. Some notable examples of thermosetting polymers include phenolic plastics, amino plastics, epoxy plastics, unsaturated polyester plastics, and organosilicon plastics [72]. The appeal of thermosetting polymers in 3D printing lies in their capacity to produce objects with exceptional structural stability and resilience.

Thermosetting polymers undergo irreversible hardening through the curing process of a viscous liquid prepolymer or resin. In contrast to unidirectional growth, active reaction sites within the polymer chain interact with adjacent chains, forming a closely interconnected polymer network. The mechanical strength, hardness, heat resistance, and chemical resistance of thermosets increase with higher crosslinking density. Curing can be achieved with heat, appropriate radiation, high pressure, and the inclusion of catalysts. Figure 4b illustrates the interconnected molecular structure of thermosetting polymers, delineating different components based on curing methods. Discussed below are a few further noteworthy explorations into the applications of thermosetting polymers in 3D printing:

Porous Carbon-Free Scaffolds for Biomedical Applications: Fu et al. [73] adopted a novel approach, combining 3D printing with ceramic conversion techniques, to fabricate porous carbon-free embedded scaffolds. These scaffolds, constructed from silicon resin infused with calcium carbonate filler under controlled atmospheres and high-temperature conditions, demonstrated impressive anti-tumor properties, particularly in bone tissue engineering. Additionally, these scaffolds exhibited the ability to enhance cell differentiation and foster bone tissue regeneration.

Clinical-Grade Photopolymerization Resins: Lin et al. [74] embarked on the development of photopolymerization resins ideally suited for 3D printing applications, leveraging an ultraviolet (UV) 3D printer. The results were impressive; the printed samples exhibited robust mechanical properties. Specifically, the bending strengths ranged from 60 to 90 MPa, the bending moduli fell within the range of 1.7 to 2.1 GPa, and the surface hardness values spanned from 14.5 to 24.6 HV. Such mechanical characteristics mirrored those of clinically used resin materials, pointing toward the significant clinical application potential of these 3D printable materials.

Strengthening Epoxy Resin with Nanomaterials: Hmeidat et al. [75] delved into the potential of integrating functional nano-clay into fiber-free reinforced epoxy resin, an endeavor realized using direct 3D printing technology. This pioneering approach yielded results that were nothing short of extraordinary. The samples produced demonstrated a remarkable strength range, measuring between 80 and 143 MPa. This strength exceeded any previously reported values for 3D-printed thermosetting composites, even those incorporating short fiber reinforcements. Their study underscores the promise of enhancing epoxy resin with nanomaterials to augment its viability in the context of direct 3D printing applications.

These investigations collectively emphasize the versatility and growing potential of thermosetting polymers in the exciting field of 3D printing. From innovative bone tissue scaffolds to precision denture production, these polymers are expanding the horizons of what is possible in additive manufacturing.

### 2.3. Hydrogels

Hydrogels are highly suitable materials for 3D printing, particularly in extrusion-based methods, due to their feasibility as ink materials [76]. The hydrogel structure consists of a 3D crosslinked hydrophilic polymer network, as depicted in Figure 4c. They can be categorized based on preparation methods into homopolymers, copolymers, and semi-interpenetrating networks (semi-IPN) [77]. Examples of homopolymers include poly(hydroxyl ethyl methacrylate) (PHEMA) and polyethylene glycol (PEG), while copolymers, such as PEG-PEGMA and carboxymethyl cellulose, incorporate two types of monomers. Semi-IPN, exemplified by acrylamide/acrylic acid copolymer, forms when a linear polymer penetrates another cross-linked network without chemical bonding. Solvents like water, ethanol, water–ethanol mixtures, and benzyl alcohol are commonly used for solution polymerization (Figure 4c) [78].

### 2.4. Elastomers

Within the realm of elastomeric materials for 3D printing, Polydimethylsiloxane (PDMS) stands out as a dominant choice, celebrated for its remarkable qualities. PDMS boasts outstanding mechanical flexibility, stretchability, chemical inertness, biocompatibility, and exceptional thermal stability when compared with other elastomers. Moreover, it maintains high chemical stability under demanding conditions of elevated temperatures and pressures. Wang et al. [22] used a direct ink writing 3D printer to craft specialized wetted surfaces using PDMS ink. They intricately patterned porous structures with varying geometric parameters and honed their design using parameter optimization. This meticulous approach resulted in the creation of sample films featuring a remarkable water contact angle of approximately 155 degrees. Notably, the porous PDMS films exhibited superhydrophobic properties and demonstrated exceptional durability, even when subjected to thermal aging.

Hollander et al. [79] explored the possibilities of using semi-solid extrusion 3D printing in conjunction with UV-LED cross-linking technology to produce PDMS drug delivery devices. Their work specifically focused on creating devices containing prednisolone. A standout aspect of this approach was its room temperature compatibility. By avoiding the need for elevated temperatures, it emerged as a promising method for manufacturing drug delivery devices designed for temperature-sensitive pharmaceuticals. Xiang et al. [80] ventured into the development of UV-curable silicone elastomers using thiol-ene photopolymerization. These materials exhibited excellent biocompatibility and found applications in skin wound healing as wound dressings. Their research highlighted the precision and versatility inherent in the thiol-ene photoreaction. It enabled the preparation of various soft structures with photolithography and the creation of three-dimensional elastic structures characterized by smooth surfaces and exceptional performance.

## 3. Smart Polymer Materials for 4D Printing

In recent years, there has been a growing interest in exploring new materials capable of exhibiting specific responses to external stimuli. This heightened interest is primarily driven by the quest to develop advanced devices, sensors, and actuators with applications spanning biomedical and electronic fields. Among the innovative materials gaining attention are smart materials, characterized by their ability to undergo changes in properties, such as shape, color, or size, in response to external stimuli like light [27], heat [25,26,34], humidity [34], or electric and magnetic fields [28,33]. This class of programmable materials introduces a unique dimension to 3D printing, referred to as 4D printing. In 4D printing, the same process as 3D printing is used, but the printed objects possess the remarkable capability to dynamically transform their shape or properties over time in response to external stimuli [19,81].

Numerous smart polymers, including SMPs, smart hydrogels, and LCPs/LCEs, have been developed; however, these materials exhibit certain limitations. Some smart polymers exhibit minimal responses over extended periods or possess limited reversibility in their transformations. Despite the need for further advancement in achieving swift and precise transformations in 4D-printed objects, the realm of 4D printing presents novel opportunities across diverse applications such as textiles [82], aerospace [18,19,83], medical industries, electronics, and robotics [18,19,84,85,86]. This section focuses on exploring smart polymers for 4D printing, particularly SMPs, smart hydrogels, and liquid crystals.

### 3.1. Shape Memory Polymers

Shape memory polymers (SMPs) have emerged as remarkable candidates for 4D printing applications due to their versatile processing capabilities, aligning perfectly with the requirements of various additive manufacturing (AM) technologies. These polymers exhibit elastic behaviors, making them well-suited for the intricate geometric transformations involved in 4D printing [8]. What sets SMPs apart is their ability to endure substantial deformation strain across a range of temperatures, allowing for dramatic and impressive shapeshifting. It is no wonder that polymeric materials have found their way into the realm of AM due to their exceptional processing attributes, making the transition to 4D printing a seamless one.

**Figure 7:**
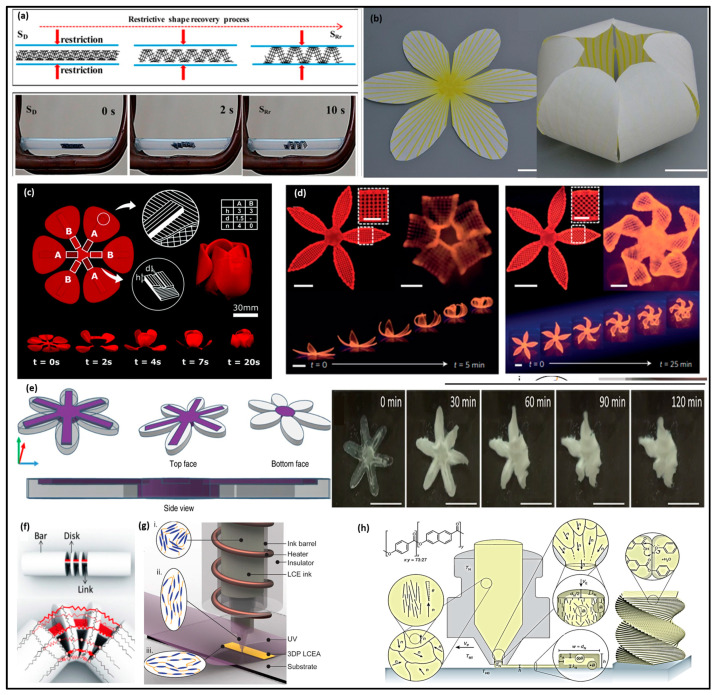
(**a**) A collection of polymer composites designed for 4D printing, featuring various innovative applications. Schematic and demonstration of a restrictive shape recovery process triggered by an alternating magnetic field [28]. (**b**) A flower-like 4D structure transforming from a flat sheet to a final flower structure [25]. (**c**) A time-lapse illustration showcasing the folding sequence of a tulip [26]. (**d**) Examples of 4D-printed flower morphologies with different bilayer directions (90°/0° and 45°/45°) [29]. (**e**) CAD models of multi-material sea stars (**left**), and demonstration of swelling over time in water (**right**) [30]. (**f**) An illustration of an initial joint and folding of bars with spring–mass systems [31]. (**g**) Illustration of the Liquid Crystal Elastomer (LCE) ink states during hot-DIW: disordered LCE ink within the barrel (**i**), aligned LCE ink as it moves through the nozzle (**ii**), and the resulting crosslinked LCE filaments post-printing (**iii**). Additionally, a visual representation of the printed LCE actuator with a meander-line print path showcases distinct elongation states corresponding to different temperatures [34]. (**h**) A schematic illustrating the printing of a core–shell microstructure with a highly aligned shell using fused filament fabrication (FFF) [35]. Figures reproduced with permission from corresponding references.

An illustrative example is a shape-changing structure under a magnetic field, depicted in Figure 7a. This structure, composed of poly(lactic acid) and magnetic iron oxide (Fe_3_O_4_) nanoparticles, undergoes remote heating under magnetic fields through hysteresis [28]. Utilizing direct ink writing (DIW) printing with UV curing facilitates the production of these shape-changing structures. While the transformation mechanisms align with other examples using thermal response, this structure exhibits fast, remotely actuated behaviors and magnetically guidable properties.

Another approach to activate SMPs involves leveraging a heat-shrinkable property, obviating the need for a shape-programming step. Figure 7b illustrates a 3D configuration transforming from a planar sheet to a final flower structure in response to temperature changes [25]. The release of internal strain in the polymer, generated during FFF, maintains the printed composite in a flat state when heated. Upon cooling to room temperature, the structure transforms into a flower configuration due to the mismatch in the coefficient of thermal expansion (CTE) between different materials. Additionally, Figure 7c presents another example using time-lapse to showcase the evolution of shape with temperature change [26]. In this instance, both geometry and printing patterns, including dimensions, the number of grooves, and active elements, were controlled during printing to induce the transformation of floral leaves into different shapes at specific times.

SMPs can be readily tailored to specific environments and applications by manipulating the crystalline content of the polymer. This fine-tuning enables the programming of critical transition temperatures, such as the glass transition temperature (T_g_) and the melting temperature (T_m_) [87,88]. A notable example of utilizing SMPs for 4D printing is the work of Ge et al., who pioneered a multi-material system for printing shape memory polymers using micro-stereolithography. Their ingenious ink formulation incorporated methacrylate-based monomers, along with essential components like the photoinitiator Phenylbis(2,4,6-trimethyl benzoyl)phosphine oxide (BAPO), Sudan I, and Rhodamine B. By adjusting the proportions of PEGDMA, BPA, DEGMA, and BMA, they could precisely tailor the T_g_ to achieve fixity control at distinct temperatures, such as 43 °C and 56 °C (Figure 7d). This precision allowed the bloom motion of their printed flower to unfold in two distinct phases [29].

Smart hydrogel composites, with their ability to swell upon water immersion while maintaining structural integrity, hold significant promise as smart materials. Their applications span diverse fields, from biomedicine including drug delivery devices [89], artificial organs, and tissue engineering [19,90], to agriculture [91,92]. In 4D printing, these hydrogel composites present an innovative method for various applications such as custom-designed sensors and robotics [19,85,86]. For example, Figure 7d illustrates biomimetic hydrogel composites in the form of functional folding flowers capable of being folded and twisted [29]. The shape transformation is governed by the anisotropic swelling behavior of the hydrogel composite, which is controlled by the alignment of cellulose fibrils along the printing directions. Another approach involves incorporating different components with distinct swelling properties within a single hydrogel structure. Figure 7e showcases sea stars that actuate via spatially controlled swelling produced with Vat Polymerization (VP) [30]. The sea stars exhibit different swelling behaviors in their center region (strain-limiting area in purple) and arms (more swellable materials in white), causing gradual curling toward the center regions over time in water. Lastly, Figure 7f depicts evolving bars composed of rigid materials (white) for bars, disks, and bottom parts of links, with the top of the links made of hydrogel (red), enabling the folding of the structure [31].

Recent advancements in the realm of 4D printing have introduced the fascinating world of cross-linked liquid crystalline polymers (LCPs) and liquid crystal elastomers (LCEs). These materials have sparked innovation in the creation of actuators and soft robotics using the process of direct ink writing (DIW) [93,94]. LCEs, in particular, are a class of materials characterized by their anisotropic nature, where their properties are finely controlled by the orientation of their molecular structure, a property that can be tailored by temperature variations [95]. Their remarkable capacity for shape transformation allows them to craft intricate structures like cones, paraboloids, and even origami-like folds [96]. Moreover, external stimuli, including heat, light, and electrical fields, rendering them dynamic and versatile, trigger the mechanical responses of LCEs.

The fascinating behavior of LCEs hinges on the arrangement of rigid molecules known as mesogens, and this behavior can be fine-tuned by altering the temperature. LCEs shift from an ordered, anisotropic state to a disordered, isotropic state at a specific transition temperature, often referred to as the isotropic transition temperature (T_i_ or T_m_) [97]. Lopez-Valdeolivas and colleagues embarked on an endeavor to create thermo-actuators using LCEs with an extrusion-based printer. In contrast to traditional methods that rely on heat-induced mesogen orientation, their innovative approach involved the extrusion process, which aligned polymer chains, effectively establishing orientational order within the mesogens. The result was an impressive 50% contraction of the printed parts when heated to 90 °C, followed by a rapid recovery to their original length upon cooling [98]. LCEs’ distinct advantage lies in their rapid recovery rates, a characteristic not commonly observed in the swelling behavior of hydrogels.

Notably, LCEs offer distinctive attributes in the world of 4D printing. For instance, Yang et al. harnessed the power of infrared (IR) light (808 nm) to fabricate, repair, and assemble carbon nanotubes (CNTs) and xLCEs for soft robotic actuators designed for low-temperature environments [99]. xLCEs boast the remarkable ability to self-heal micro-cracks through photo-healing, ensuring a sustainable and efficient material life cycle. Furthermore, CNT-xLCEs are manufactured with transesterification processes carried out at temperatures exceeding 180 °C. While many of the mentioned shape memory polymers (SMPs) thus far have been thermoplastic in nature, designed to regain their shape through temperature-induced transitions involving the glass transition temperature (T_g_) and melting temperature (T_m_), there exists a realm where robustness is paramount. In scenarios where extreme and harsh environments demand chemically and thermally stable materials, thermoset plastics have often been the choice due to their strong chemical covalent cross-links, ensuring excellent chemical and thermal stability.

Figure 7g depicts variations in the morphologies of photopolymerizable liquid crystal elastomer (LCE) ink during direct ink writing (DIW) and the diverse elongation of the printed LCE based on the aligned printing path, depending on the temperature relative to the nematic–isotropic transition. In another extrusion printing process using solid filaments, liquid crystal polymers (LCPs, Vectra A950) [35], as shown in Figure 7h, exhibit a notable alignment of nematic domains through the nozzle due to shear forces during extrusion. Despite the initial alignment, the printed filament tends to lose its orientation and solidifies from the surface, resulting in a core–shell microstructure with a highly aligned shell. The mechanical properties of this printed hierarchical structure can be further strengthened with thermal annealing, facilitating chemical crosslinking of chain ends between filaments.

**Figure 8:**
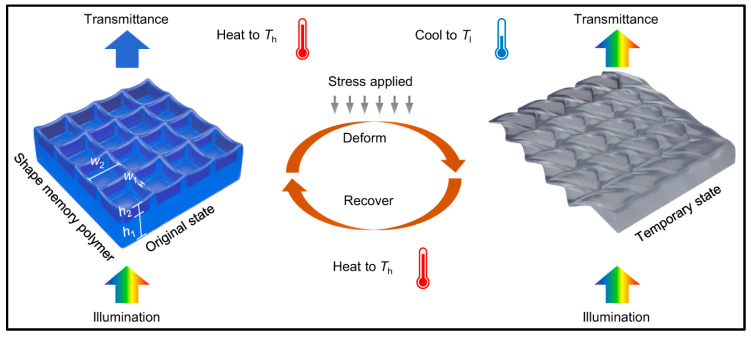
Illustration depicting the color and shape transformation of a constituent nanostructured element in the “invisible ink” 3D printed with a shape memory polymer. The as-printed structures feature upright grids on the left, acting as a structural color filter that selectively transmits a limited wavelength range of visible light. Deformation of the structures at elevated temperatures flattens the nanostructures on the right, resulting in a colorless state, which persists as an invisible state after cooling to room temperature. Heating restores both the original geometry and the color of the nanostructures, showcasing a submicron demonstration of 4D printing. Figure reproduced with permission from [100].

A study conducted by Zhang et al. demonstrated a fascinating correlation between color transmittance and the nanostructure of a 4D-printed shape memory polymer (SMP) [100]. Using microstereolithography (MPL), the group 4D printed submicron-scale grids using VeroClearTM. When these grids were illuminated with white light, they selectively transmitted a limited range of wavelengths due to the differential scattering of the spectrum by the grid (see Figure 8). The dimensions of the grid determined which wavelengths could pass through. Upon programming the structure, i.e., heating it above the glass transition temperature (T_g_) to 80 °C, distorting it, and cooling it, the structure became transparent to all wavelengths, achieving an “invisible” state. Reheating above T_g_ restored the nanostructure to its as-printed state, allowing only a specific range of wavelengths to pass through once again.

**Figure 9:**
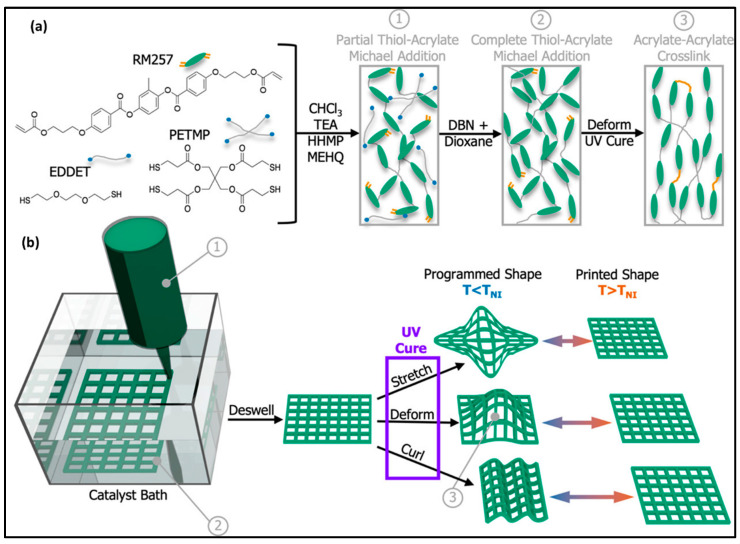
Schematic illustration of the reactive 4D printing process and shape programming of liquid crystal elastomers (LCEs). (**a**) Depiction of the LCE synthetic scheme, highlighting the network-forming components and three distinct reactive steps during the fabrication process. (**b**) Schematic representation of the 4D printing of LCEs, involving printing in a catalyst bath, followed by deformation and UV curing for shape programming. The resulting LCE demonstrates reversible shape changes between the printed and programmed structures when heated and cooled, respectively. Figure reproduced with permission from [101].

LCEs have also been successfully 3D printed in their isotropic state, providing the user with the flexibility to manually determine the nematic director of the mesogens post-printing. The resulting 4D-printed smart LCE object demonstrates the capability to switch between the high-order post-printing programmed shape and the low-order shape determined by the printing process. In the study by Barnes et al., a specially formulated LCE ink was used, comprising RM257 mesogens and chain extenders 2,2′-(ethylenedioxy)diethanethio (EDDET) and pentaerythritol tetrakis (3-mercaptopropionate) (PETMP) (Figure 9a) [101].

The ink was partially crosslinked and DIW-printed into a catalyst bath, where further crosslinking occurred, resulting in a 3D LCE structure without a defined director (Figure 9b). The printed object contained residual unreacted acrylate functional groups. By stretching the object in a specific direction, a nematic director was established parallel to that direction, and UV light was applied to crosslink the residual acrylate groups, setting the programmed shape. Upon heat treatment to the transition to nematic isotropy (TNI) at 75 °C, the mesogens relaxed, and the programmed structure reverted to the original printed structure. This shape evolution could be reversed by cooling the object.

**Figure 10:**
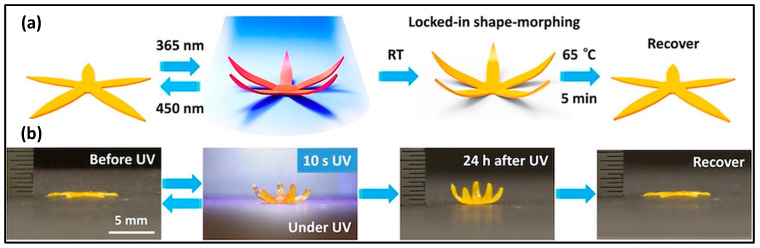
(**a**) Illustration and (**b**) photographs depicting the photo-switchable deformations of a flower-like actuator 4D printed with light-sensitive LCE. Under 365 nm UV light, the flower curled, and the process was reversed with 450 nm blue light. After UV irradiation, the curled shape was stabilized at room temperature via the formation of UPy crosslinks. The original flat shape was recovered by heating to 65 °C, breaking the crosslinks, and allowing the LCE to entropically switch back to the isotropic state. Figure reproduced with permission from [104].

Azobenzene moieties undergo isomerization from the trans to cis state upon UV irradiation, causing an expansion of the polymer matrix. This phenomenon has been utilized in 4D printing to create light-responsive actuating silicone bilayers [102]. In the case of 4D printable LCEs, azobenzene moieties contribute to light responsiveness at ambient temperature, inducing stress and mesogen misalignment. Ceamanos et al. demonstrated the 3D printing of an azobenzene-containing LCE strip that contracted under UV light, lifting a weight, and recovered its initial length under blue light [103]. However, spontaneous relaxation of azobenzene moieties over time posed stability challenges. Lu et al. addressed this issue by incorporating 2-ureido-4[1H]-pyrimidinone (UPy) physical crosslinkers into the LCE ink, allowing the structure to change shape via photoisomerization of azobenzene moieties under UV light. The shape was then stabilized by the reformation of UPy crosslinks when the UV light was removed [104], as shown in Figure 10.

### 3.2. Self-Healing Polymer Materials

In the context of 4D printing, self-healing materials present a remarkable capability. These materials have the unique ability to adapt and evolve over time, offering increased longevity and reliability to printed components in a variety of applications, including electronics and healthcare monitoring. Self-healing materials are a valuable addition to the toolkit of 4D printing, offering the potential for more resilient and long-lasting printed structures. By integrating self-healing properties into 4D printing materials, engineers and designers can create structures that can endure harsh environments, maintain their functionality over extended periods, and reduce the environmental impact associated with the disposal of damaged items. This remarkable feature of self-healing materials represents a significant advancement in 4D printing technology, with widespread potential across multiple industries and applications. Self-healing materials operate by autonomously initiating the repair process when defects like cracks or scratches occur. This spontaneous healing occurs as the material fills the void created by the defect with fresh material. The ultimate goal of an ideal self-healing material is to fully restore the original mechanical and chemical properties of the pristine material [105]. These self-healing materials can be broadly categorized into two main types based on their healing mechanisms. Autonomous materials are capable of immediately commencing the healing process upon sustaining damage. This spontaneous repair is facilitated through mechanisms like reversible hydrogen bonds, non-covalent bonds, or the release of self-healing substances that are embedded within the host material and triggered by damage [106]. Ton-autonomous materials, in contrast, require external stimuli to initiate the healing process. These stimuli can include factors such as exposure to UV light, application of heat, or mechanical activation. These external influences are necessary to trigger repair [106].

#### 3.2.1. Microvascular Self-Healing Mechanisms

**Figure 11:**
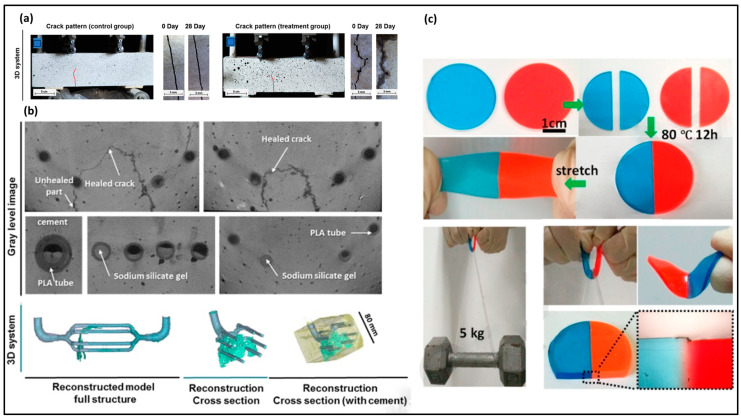
(**a**) Representation of a vascular beam crack pattern and microscopic imagery documenting the healing process following a 4-point bend [109]. (**b**) A CT grey level image unveiling the concrete matrix, cracks, PLA vascular tubes, and sodium silicate self-healing agent. The 3D system was reconstructed to illustrate the vascular mechanisms, with the yellow area symbolizing the cement, violet sections representing the PLA vascular structure, blue section representing sodium silicate gel, and the green area depicting the gel filling in cracks [109]. (**c**) Depictions of colored self-healing polyurethane samples that were cut, connected, and healed at 80 °C for 12 h. The healed sample is showcased while being stretched and supporting a 5 kg weight [111]. Figures reproduced with permissions.

Self-healing mechanisms using microvascular systems incorporate a network of channels within a material, filled with a healing agent, traversing the material’s matrix. Upon the occurrence of a crack or damage, capillary action initiates the release of the healing agent into the damaged area, where it solidifies to seal the crack. This concept draws inspiration from the natural arterial system [107,108]. Li et al. [109] introduced an innovative biomimetic 3D vascular design inspired by nature for self-healing cementitious systems (Figure 11a–c). The 3D printed design featured a polylactic acid (PLA) vascular network produced on an Ultimaker^®^ 3D printer (FFF). This network incorporated the healing agent sodium silicate. In instances where a crack formed in the cement matrix, the vascular design adhered to Murray’s law for circulatory blood volume transfer, thereby facilitating the healing process. Another approach was taken by Wu et al., who developed a microvascular network using a fugitive ink and hydrogel reservoir to create a self-healing printed material tailored for applications in healthcare microvascular devices [110].

Balancing the characteristics of printable inks with multiple functionalities is a delicate task, as it involves finding the right balance between printability properties and functional properties. In this instance, thiol groups play a role in the photocuring process, while disulfide groups contribute to self-healing properties. Li et al. also documented the printing of a photocurable elastomeric material using DLP (Figure 11c) [111].

#### 3.2.2. Encapsulation Self-Healing Mechanisms

Encapsulation-based self-repair mechanisms utilize micro- or nano-sized capsules filled with a healing agent, such as a polymer or catalyst. Each capsule is shielded by a case or coating made from an inert material to prevent interference with the bulk material. This ensures that the healing process only commences when damage occurs, not prematurely. The capsule’s shell is designed to be fragile, enabling easy rupture upon damage. These capsules are distributed throughout the material’s matrix to ensure a rapid release of the healing agent upon rupture. The healing agent inside the capsules should have low viscosity to facilitate capillary action into the damaged area. For efficient healing, the healing agent should polymerize quickly at room temperature without shrinkage [112]. Davami et al. used SLA 3D printing to create structures that entrap photocurable resin within unit cells, acting as a self-healing agent. When damage occurs, the self-healing agent flows out of the cell through capillary action to the damaged site, where it is cured using UV light. Davami et al. reported a healing efficiency of 52% based on fracture toughness [113].

#### 3.2.3. Autonomous Self-Healing Polymers: Supramolecular Polymers

Most polymers typically require the presence of a host network containing a self-healing agent, such as capsules or vascular systems, while supramolecular polymers constitute a distinct class of materials. Characterized by reversible non-covalent bonds, these polymers can reform and repair after being cleaved [114]. The inherent self-healing properties of supramolecular polymers distinguish them, allowing for repeated healing without depleting a host healing agent. Often described as “solid-liquids,” these polymers consist of associated groups typically covalently bonded to chain ends or side chains of a polymer. These groups bring together liquid-like polymers into a network of non-covalently cross-linked polymers, exhibiting plastic behaviors. The reversible reforming process of supramolecular polymers involves non-covalent crosslinks like π-π stacking, hydrogen bonds, host–guest interactions, ionic interactions, and metal coordination. Evaluating the effectiveness of restoring a self-healing material involves examining various properties, such as mechanical, electrical, and thermal properties, before and after healing. This assessment, known as healing efficiency, is calculated using the ratio of these properties before and after healing [114].

## 4. Hybrid Materials for Printing

### 4.1. Polymer–Metal Composites

#### 4.1.1. Three-Dimensional Printing of Polymer–Metal Composite Feedstocks

**Figure 12:**
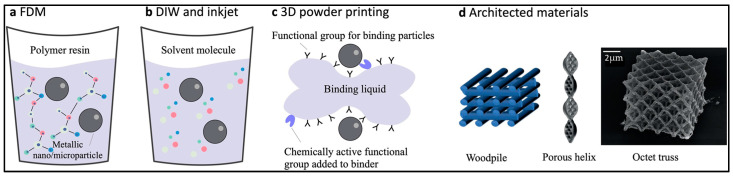
Deployment of metallic nano/microparticles spans various additive manufacturing techniques: (**a**) integration of polymer–metal filaments in fused filament fabrication (FFF), (**b**) utilization of water-based metal inks in direct ink writing and inkjet printing, (**c**) application of UV-curable metal resin in stereolithography and 3D powder printing, and (**d**) illustration of characteristic metallic structures designed for catalyst applications. On the right side: portrayal of the nickel nanolattice octet truss post-pyrolysis [115,116]. Figures reproduced with permission.

Polymer–metal composites involve the amalgamation of metal particles with a liquid polymer resin, typically an unsaturated polyester, in the form of water-based substances exhibiting heightened viscosities. In Figure 12, diverse methodologies are depicted for crafting resilient structures utilizing polymer–metal hybrid materials across various 3D printing techniques [115]. In specific environments, such as high-temperature settings, metal filaments and water-based metallic ink can be extruded through a nozzle, subsequently solidifying rapidly upon cooling or further sintering in a furnace (Figure 12a,b). Photo-hardening polymers, comprising liquid polymer resin, a crosslinking agent, an initiator, and a photosensitizer, can be cured with UV light, wherein the binding liquid facilitates bonding metallic particles together in powder form (Figure 12c). Objects 3D printed with metallic nano/microparticles have gained extensive application in the creation of catalytic and mechanically robust structured materials (Figure 12d).

In addition to utilizing polymer–metal feedstocks, the synthesis of metal–polymer composites can be achieved with a distinct 3D printing approach—constructing a metal framework and subsequently infusing it with polymers. This method brings forth notable advantages, notably heightened conductivity in functional applications and superior mechanical properties. The synergistic effect of the composite material disperses energy efficiently, substantially enhancing fracture toughness, particularly in scenarios where traditional 3D-printed parts exhibit imperfections from the manufacturing process [117]. The stiffness balance between the soft and hard phases can be finely tuned to bolster fracture toughness in structured materials. Epoxy stands out as the preferred base material for polymers due to its commendable strength and straightforward processing. Bapari et al. [118] conducted insightful studies that illustrated the pivotal role of the soft phase in fortifying rigidity in metal–particulate polymer composites. Meanwhile, Li et al. [119] delved into the intricacies of interpenetrating phase composites (IPCs) within selectively laser-melted (SLM) metallic micro-lattices. Their experiments and simulation models unveiled that achieving uniform deformation in reinforced struts demanded more energy than the buckling of freestanding struts.

The amalgamation of polymers and metals in the fabrication of objects unveils captivating mechanical and functional properties. Despite the conventional belief that metal–polymer blends may not be ideal for swiftly manufacturing objects with high mechanical strength, significant research efforts have been devoted to exploring innovative materials. Fafenrot and colleagues [120] noted the impact of polymer–metal feedstocks containing bronze, revealing a noticeable reduction in mechanical properties. Zhu and his team presented a groundbreaking creation—hierarchical nanoporous gold with meticulously engineered nonrandom macro-architectures using DIW (direct ink writing) and dealloying techniques. The resulting nanoporous metals showcased significantly enhanced mass transport properties and reaction rates for both liquids and gases. Furthermore, Vyatskikh and collaborators pioneered a lithography-based process for producing intricate 3D nano-architected metals boasting approximately 100 nm resolution. This breakthrough transcended the limitations of existing methods, which typically offer resolutions in the range of 20 to 50 μm for 3D-printed metals.

#### 4.1.2. Surface Coating of Metals on 3D-Printed Polymers

Nature demonstrates its mastery in developing robust and flexible materials through the creation of intricate, multi-scale hierarchical structures. This has led to the development of a unique class of biological layered lattice materials, featuring hierarchical levels ranging from the microscale to the nanoscale. These mechanical metamaterials, characterized by lightweight properties, derive their high mechanical strength from the well-established principle that “smaller is stronger”, rooted in the effects observed at the nanoscale and optimized geometric arrangements. What sets these materials apart is their ability to exhibit unconventional mechanical behaviors, such as twisting or expanding in all directions under uniaxial compression—feats unattainable with traditional materials.

**Figure 13:**
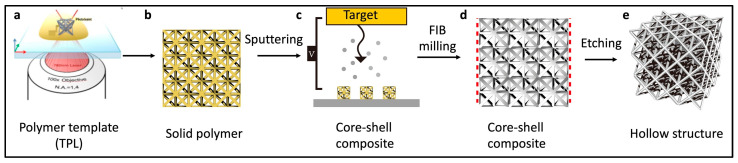
Illustration depicting the manufacturing procedure for creating hierarchical polymer-metal composites: (**a**–**c**) application of metal coatings on 3D-printed polymers, and (**d**,**e**) production of hollow-beam metallic architected materials. Figure reproduced with permission from [12].

**Figure 14:**
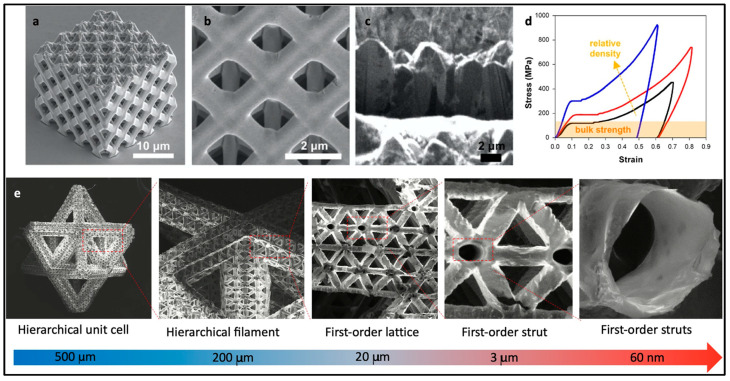
Hierarchical polymer–metal composites from mesoscale to microscale: (**a**,**b**) creation of core–shell Cu meso-lattices using two-photon lithography followed by electroplating, (**c**) cross-sectional milling of Cu film with a focused ion beam, (**d**) stress–strain curve of core–shell Cu lattice demonstrating higher compressive yield strengths compared with its monolithic bulk counterparts [121], and (**e**) construction of a hierarchical metamaterial composed of Ni micro-lattices with periodic hollow tubes, spanning critical feature length scales across seven orders of magnitude [122]. Figures reproduced with permission.

In the realm of architected materials, the focus of many studies is to replicate the exceptional characteristics found in natural structural materials. One approach involves the creation of core–shell composites, featuring soft polymer cores enveloped by layers of ultra-strong but brittle metallic or metallic glass (MG) coatings (Figure 13a–c). Numerous instances of 3D-printed trusses showcase feature sizes spanning various orders of magnitude, from tens of millimeters to hundreds of nanometers (Figure 14). For example, Cu octet lattices have achieved specific strengths of approximately 400 MPa, exceeding the yield strengths of bulk counterparts by an impressive 80% (Figure 14a–d) [121]. The remarkable strength of these core–shell metallic lattices is attributed to a size effect, with the material consisting of single-crystalline metal featuring submicron dimensions. These findings offer valuable insights into and a deeper understanding of the mechanical properties achieved with the utilization of hierarchical structures and innovative material design.

Thus, combining metals and polymers in 3D printing opens up opportunities to create materials with exceptional properties and characteristics. Researchers have explored various methods, such as nanoporous structures and core–shell composites, to enhance the mechanical and functional aspects of these hybrid materials. These innovations have the potential to impact fields like aerospace and medicine where advanced materials are in demand. The primary objective of the current research is to empower hierarchical polymer–metal composites to achieve remarkable levels of stiffness, strength, and toughness while maintaining very low densities. The challenge lies in finding the right balance between structure-dominated buckling failure and metal-dominated brittle failure. For instance, polymeric octet lattices with 1 μm strut diameters coated with a 10 nm NiB metallic film exhibited delayed buckling.

This elastic buckling allowed for significant rotation of the nodes, resulting in ductile-like deformation. On the other hand, thicker coatings, such as 100 nm, led to brittle failure [123]. The susceptibility to brittle failure also depends on the type of metal and the specific architecture. When utilizing High-Entropy Alloys (HEAs), the film thickness can be increased to 50 nm, overcoming the strength–recoverability trade-off [124]. While thicker coatings can better contain the polymer core, the optimal coating thickness was found to be in the range of 14–50 nm to prevent catastrophic failure. To leverage the “smaller/thinner is more ductile” size effect, it is crucial to devise innovative architectures and explore the potential of strong yet ductile metallic films.

One common approach for architectural design involves etching away the plastic core to obtain a pure, ultra-lightweight metal (Figure 13d,e). A hollow-beam nickel lattice, characterized by a nearly constant specific strength, demonstrated a high tensile elasticity of over 20%, which was unattainable for the individual metallic components [122] (Figure 14e). Future research endeavors should address both scientific and technological challenges, including the trade-off between strength and ductility [125], as well as the trade-off between speed and resolution [43].

In addition to pursuing high strength-to-density ratios and pushing the boundaries of material properties, emerging materials with innovative designs, such as chiral mechanical metamaterials and materials featuring tunable mechanical properties and programmable stimulus responses, are beginning to gain attention. To use this new category of materials in multifunctional engineering applications, it is essential to develop physical models or theories capable of capturing the architectural complexity and propagating the exceptional properties from the nano/microscale to the macroscale.

### 4.2. Polymer–Ceramic Composites

#### 4.2.1. Three-Dimensional Printing of Polymer–Ceramic Composite Feedstocks

The primary constituents used in the 3D printing of polymer–ceramic composites include ceramics, resins, or a blend of both [126]. These materials are divided into two categories based on their form: liquid or semi-liquid inks and pastes and fine ceramic particles. Inks and pastes are a mixture of liquid or paste resin with finely dispersed ceramic particles. Their usage in 3D printing is determined by their viscosity and can include methods such as photopolymerization, inkjet printing, and extrusion [127]. Fine ceramic particles, on the other hand, are fused together either with a powder fusion and melting process, often using a heat source like a laser, or by using viscous liquid binders like non-hydroxyl resin and liquid paraffin. This kind of feedstock is suitable for 3D printing techniques such as binder jetting (BJ), selective laser sintering (SLS), or selective laser melting (SLM) [127].

#### 4.2.2. Multi-Material Printing of Polymer–Ceramic Composite Structures

High strength and toughness are fundamental requirements for a wide range of engineering materials, but they often exhibit a mutually exclusive relationship [128]. For example, enhancing the strength of steel using cold machining typically results in a loss of toughness. Similarly, engineering ceramics surpass metals in hardness and strength but are limited by their relatively low toughness values [129]. Recent research has highlighted the remarkable combinations of strength and toughness found in natural bio-ceramics like bones or shells [130,131]. Inspired by these natural ceramics, various fabrication techniques have emerged to replicate bio-ceramics, including ice templating, layer-by-layer deposition, self-assembly, and rapid prototyping [132,133,134]. These studies illustrate how the trade-off between toughness and strength can be addressed by creating architected composites with well-organized microstructures.

**Figure 15:**
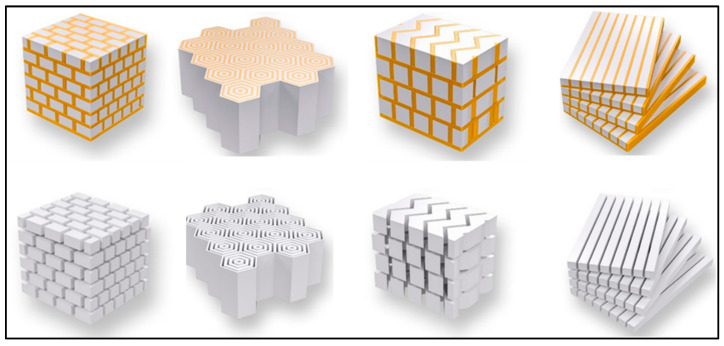
Emulating the structures of nacre [141], bone osteon [138], dactyl club [140], and conch shell, from left to right. The top-row representations of these structures incorporate rigid ceramic skeletons (depicted in white) and flexible polymer phases (depicted in yellow). The bottom row showcases the skeletal framework corresponding to the left. Figures reproduced with permission.

Recently, the field of 3D printing has opened up new possibilities for fabricating microstructures with hierarchical or arbitrary geometries, utilizing a bottom-up approach similar to processes found in nature. This has led to a significant advancement in the study of bio-ceramics [125,135,136,137]. For example, by using multi-material 3D printers, researchers have been able to create staggered microstructures that closely resemble the architecture of bones [136]. This innovative approach resulted in a remarkable increase in fracture energy, exceeding the previous levels by tenfold. Moreover, 3D printing techniques have been used to replicate the intricate structures found in natural materials such as nacre, bone osteons, dactyl clubs, and conch shells [138,139,140,141], as illustrated in Figure 15. These endeavors aim to unravel the precise designs found in bio-ceramics. In specific cases, structures resembling dactyl clubs have been produced, exhibiting a J-shaped R-curve (crack growth resistance curve), while others have displayed Γ-shaped R-curves. A J-shaped R-curve is associated with a significantly larger critical energy release rate, making it advantageous for halting crack propagation. In contrast, a Γ-shaped R-curve is characterized by a higher critical failure stress, which effectively hinders crack initiation [142]. Additionally, researchers have explored the manipulation of magnetic fields to guide the orientation of magnetic platelets, forming intricate patterns such as concentric and layered designs [142]. As a result, 3D printing methodologies offer a versatile platform for creating complex designs to investigate the geometric influences on mechanical performance, particularly toughness, within the realm of bio-ceramics.

**Figure 16:**
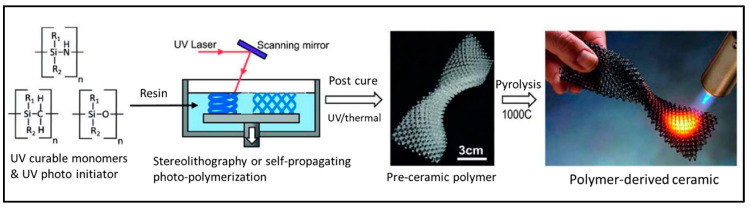
The process of 3D-printed polymer-derived ceramics. The depiction of the novel process where polymer-derived ceramifiable monomers, like silicon oxycarbide, are 3D printed and polymerized with UV light, followed by high-temperature sintering. Figure reproduced with permission from [143].

A highly promising technique in 3D printing involves the utilization of polymer-derived ceramifiable monomers, notably silicon oxycarbide. These specialized monomers are used in the 3D printing process, initially taking shape through layering and then undergoing polymerization using UV light. Following this stage, they are subjected to high-temperature sintering, a critical step that transforms them into intricate and high-quality ceramic lattices [143]. This entire process is depicted in Figure 16, which provides a visual overview of the 3D-printed polymer-derived ceramic production journey.

One notable advantage of polymer-derived ceramics is that they eliminate the need for extensive post-processing to remove the organic binder, which is often required when using ceramic fillers. This streamlined production process results in reduced manufacturing times, making it an efficient choice. The ceramic stereolithography (CSL) yields exceptionally smooth surface finishes, enhancing the overall quality of the ceramic structures it produces. However, it is essential to note that while CSL offers distinct advantages, it is considered relatively expensive due to the specialized equipment and materials required. Additionally, the selection of available materials for this technique is somewhat limited, which is viewed as one of its main drawbacks [144].

### 4.3. Polymer–Metal–Ceramic Composites

The role of printing materials is increasingly pivotal in expanding the applications of 3D printing technology. In recent years, there has been a growing trend to elevate the proportion of composite materials used to meet the intricate demands of printed products in various industries. Commonly used composite materials in 3D printing encompass combinations of metals with ceramics, polymers with ceramics, and polymers with metals. However, with the continuous evolution of “intelligent manufacturing” centered around 3D printing, the material composition of printed components often necessitates the fusion of metals, ceramics, and polymers to fulfill diverse performance criteria [145]. Although research into the domain of composite 3D printing involving metals, ceramics, and polymers is still in its nascent stages, there are emerging reports regarding its application in biomedical, electronics, surface engineering, and other domains. There are primarily two methods for printing composite materials: layered powder printing and mixed powder printing.

**Figure 17:**
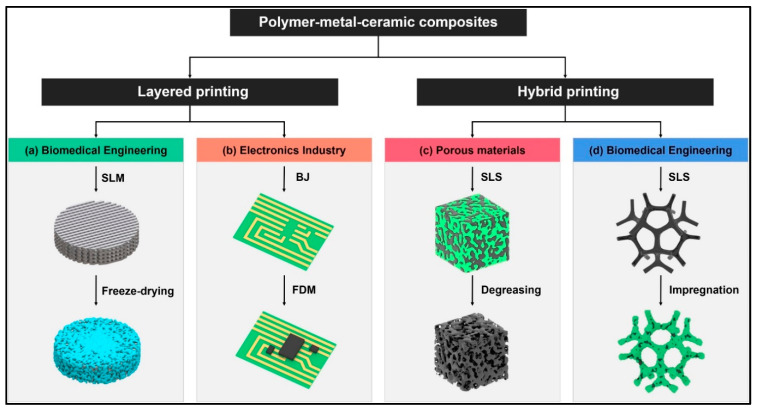
Potential applications and future advancements in polymer–metal–ceramic composites. Figure reproduced with permission from [12].

In layered printing, materials are progressively printed to create intricate structures. For instance, in the domain of bone tissue repair that requires load-bearing capabilities, porous scaffolds are commonly printed using metal materials, while a bio-ceramic–polymer coating is applied to the surface of the structure (Figure 17a) [146]. Such bone tissue structures composed of metals, ceramics, and polymers offer advantages such as mechanical compatibility with natural bone, corrosion resistance, and biocompatibility. In the electronics field, products like printed circuit boards (PCBs) often demand a combination of metal, ceramic, and polymer materials for their production. PCBs are initially printed with metal materials, and subsequent components like capacitors are printed directly onto PCBs using ceramics and polymer materials (Figure 17b).

In mixed powder printing, metal/ceramic/polymer powders are typically prepared with mechanical mixing. During the selective laser sintering (SLS) process, a polymer material is removed, leaving behind a porous structure (Figure 17c). Additionally, these initial porous structures formed using SLS can be infiltrated with another material to enhance their density and mechanical properties (Figure 17d). For the repair of non-load-bearing bone tissue, printing often involves the use of calcium phosphate and hydroxyapatite (HA) composite biocompatible polymers [147]. Furthermore, magnesium can be uniformly integrated into artificial bone structures during the printing process, thereby enhancing osteoinductive activity and osteoacusis performance [148].

In a multitude of industries, especially in the fields of electronics and medicine, certain critical components or products require a combination of properties associated with metals, ceramics, and polymers. The traditional methods of preparing such components are typically cumbersome and expensive. However, 3D printing technology enables the creation of complex components in a single step, eliminating the need for multiple processes. This approach offers significant advantages due to its cost-efficiency and increased speed. Nevertheless, it is important to acknowledge that the current state of 3D printing technology cannot fully support widespread applications in the production of multi-material components, and there is a relative scarcity of printable materials in this domain. Therefore, further research is required to advance this area of study.

## 5. Three-Dimensional/Four-Dimensional Printing and Biomedicine

In recent years, the intersection of engineering and biomedical sciences has led to significant advancements in 3D and 4D printing technologies. These technologies have been increasingly utilized in the development of medical devices and drug delivery systems [149,150,151]. Bioprinting, an innovative and emerging technology of additive manufacturing, has revolutionized the biomedical sector by printing 3D cell-laden constructs in a precise and controlled manner for numerous clinical applications. This approach uses biomaterials and varying types of cells to print constructs for tissue regeneration, e.g., cardiac, bone, corneal, cartilage, neural, and skin. Furthermore, bioprinting technology helps to develop drug delivery and wound healing systems, bio-actuators, bio-robotics, and bio-sensors [149,150,151].

More recently, the development of 4D bioprinting technology and stimuli-responsive materials has transformed the biomedical sector with numerous innovations and revolutions [149,152]. The term 4D printing was coined to indicate the combined use of additive manufacturing, smart materials, and careful design of appropriate geometries. In this review, we report the recent progress in the design and development of smart materials that are actuated by different stimuli and their exploitation within additive manufacturing to produce biomimetic structures with important repercussions in different but interrelated biomedical areas [152]. The advent of 3D and 4D printing technologies has also led to the creation of personalized drug delivery systems [150,153,154]. These systems can be tailored to meet individual needs, addressing the challenges associated with the manufacture of pharmaceutical systems [151,153]. This has resulted in many concepts of pharmaceutical devices and formulations that can be printed and, possibly, tailored to an individual [150]. Thus, the bio-related applications of 3D and 4D printing are vast and rapidly expanding. Future research in this area promises to bring about even more innovative solutions for personalized medicine and healthcare [149,150,151].

In a comprehensive exploration of smart polymers, Huang et al. [155] delve into thermally responsive soft materials that adapt to environmental changes. Their review elucidates the design, characterization, and progress of these polymers, particularly in medical devices, cell therapy, and 3D printing for precision medicine. Another contribution comes from Pineda-Castillo et al. [156], who discuss state-of-the-art endovascular devices and the potential of shape memory polymers (SMPs) in addressing limitations related to complete occlusion, focusing on their application in Intracranial Aneurysm (ICA) embolization. Uboldi et al. [157] present a lab-scale film-coating process for self-expanding rod-shaped Polyvinyl Alcohol (PVA)-based devices fabricated with Hot Melt Extrusion (HME) and fused filament fabrication (FFF), offering precise control over diffusion and transport processes. In the realm of 4D bioprinting, Afzali Nanizetal [158] explores recent developments in smart materials and their applications in developing biomimetic structures with actuation capabilities, impacting pharmaceutics and biomedical research. Lastly, Sirawit et al. [159] propose a novel approach to manipulating the swelling kinetics of hydrogels with a diffusion-path architecture design, offering a unique perspective on 4D-printed hydrogel actuators.

## 6. Future Perspectives of Polymer AM and Challenges Ahead

In this section, we delve into the ongoing evolution of polymer 4D printing, examining its future trajectory. We identify five key areas critical for its advancement, categorized into smart materials and printing techniques. Within the smart material domain, we focus on particle–polymer composites (PPCs), “living” polymers, and polymers endowed with self-healing/welding capabilities. In the realm of printing techniques, our attention shifts to multimaterial polymer 4D printing and microscale polymer 4D printing.

In summary, this comprehensive examination reveals the significant strides made in 3D printing techniques and the pivotal role of functional polymers in reshaping the manufacturing landscape. The persistent refinement of 3D printing, with resolutions now approaching the nanoscale, paves the way for the production of high-performance products characterized by meticulously optimized structures and functions, spanning across diverse application fields [43,160]. Furthermore, by actively engineering the chemical and physical properties of polymers, we can unleash shape-changing capabilities and exercise precise control over the motion of printed products. While additive manufacturing technologies have matured and unlocked novel possibilities in a multitude of domains, they are accompanied by several challenges that merit attention in forthcoming research. These challenges serve as indispensable guiding principles for further studies, offering the potential for pioneering advancements in polymer development and applications. Despite these challenges, the future of polymer AM holds great promise. The continual development of materials, the improvement in material properties, and advancements in printer technology are expected to drive the evolution of polymer AM. This technology is on a trajectory to revolutionize manufacturing processes, expand applications, and enable the creation of innovative, high-performance components that cater to a wide range of industrial needs.

### 6.1. Material Diversity

The array of printable materials remains somewhat constrained due to the specific requirements of 3D printing, encompassing aspects like rheology and melting points. For example, widely used materials with low melting points and suitable viscosities, as seen in fused filament fabrication (FFF), often result in less-than-ideal mechanical and chemical properties of the final products. To meet the stringent requirements of aerospace, automotive, and structural applications, it is imperative to delve into the development of engineering-grade polymers. It is equally important to extend our perspective by considering a vast spectrum of functionalities in polymers and polymer composites for 3D printing. This expansion can take inspiration from the ingenuity of biological systems and biomaterials, spurring materials development, functional innovation, and novel structural designs.

### 6.2. Multi-Material and Multi-Scale AM for Various Applications

The critical drive toward multi-material and multi-scale manufacturing techniques is essential to simultaneously control material composition, ratio, functions, and internal architecture at micro- and nanoscales. Applications spanning biology, electronics, and robotics necessitate the use of multiple materials across different scales to enable complex movements or specific reactions. The evolution of multi-material and multi-scale AM represents a transformative force, reducing material waste and streamlining assembly steps across various industries. However, some 3D printing techniques encounter challenges in terms of printing with multiple materials. Innovative approaches, including the introduction of rotating vat carousels and the incorporation of microfluidic systems, have been developed to tackle these challenges [161]. Despite significant strides in composite printing, limitations persist, such as height restrictions and the difficulties encountered when fabricating products with a high loading of reinforcements, primarily due to the existing printing constraints. Looking ahead, the emergence of hybrid AM appears promising in addressing a multitude of unique customization needs, with the tantalizing prospect of 3D printing robots capable of walking off the printing plate.

### 6.3. Improving AM Processes for High-Quality Prints

Additive manufacturing processes, though revolutionary, introduce certain defects within printed products, impacting their mechanical properties and causing discrepancies between a digital model and a physical object. Notably, void formation remains a significant challenge in techniques such as powder bed fusion (PBF) and binder jetting (BJ). These voids materialize due to insufficient bonding between melted materials and binders. Additionally, addressing weak interlayer bonding within manufactured parts is crucial to mitigate anisotropic and subpar mechanical properties. These issues can also lead to delamination between layers. Ongoing research is dedicated to overcoming these hurdles, using tactics like introducing fillers and harnessing laser or infrared heating between printed layers. Emerging techniques, including volumetric manufacturing, hold substantial promise in surmounting these limitations [162]. Moreover, it is essential to integrate in situ monitoring and post-inspection processes to bolster quality control.

### 6.4. High-Throughput and Scalable Manufacturing

The speed of various 3D printing technologies during the actual printing phase remains a point of concern. The subsequent post-processing steps further diminish throughput and scalability. The industry’s response involves deploying robot arms in fused filament fabrication (FFF) to circumvent the limitations posed by traditional printing plates, allowing for the production of large-scale products. Meanwhile, the application of chemical or water-soluble support materials in FFF simplifies post-processing by reducing the time spent on support cleaning. However, certain 3D printing methods are inherently limited when it comes to building size, and rapid printing of large parts can generate excess heat, leading to distortion in the final products. Recent research endeavors seek to bolster productivity by managing heat generation using mobile oil [163]. Innovative printing concepts, exemplified by volumetric AM, have begun to emerge as an enticing trend [162]. Furthermore, addressing inconsistencies in printing quality calls for the integration of feedback systems powered by machine learning, ensuring superior repeatability in scalable manufacturing [164].

### 6.5. Sustainability

The pursuit of a sustainable future necessitates a concerted effort to reduce waste within the realm of 3D printing. The journey toward environmental friendliness is multifaceted. It involves improving biodegradable polymers while transitioning from oil-based feedstocks to bio-based compostable plastics [23]. Additionally, managing failed prints and end-of-life products with recycling holds the promise of reducing material costs and minimizing the demand for frequent parts resupply in supply chains. The recyclability of thermoplastics stands as a notable advantage over thermosets due to the limited degradation of polymer chains. Thermoplastics are more easily recyclable compared with thermosets because they experience little to no degradation of the polymer chain when melted down. In thermoplastics, weaker interactions between polymer chains are broken, while in thermosets, the bonds between monomers deteriorate with each reuse, making it challenging to recycle thermoset polymers. An example of thermoplastic recycling is the processing of polyethylene terephthalate bottles into filaments for 3D printing [165]. These recycled filaments demonstrate comparable tensile strength and elongation, making them capable of replacing commercial filaments. Additionally, research is ongoing in other 3D printing methods, such as powder bed fusion (PBF) and Vat Polymerization (VP), to explore the reuse of unused materials, such as powder and resin, respectively.

### 6.6. Safety Concerns

The growing interest in and adoption of 3D printing have inevitably brought to the fore various safety concerns. Polymer-based AM processes have the potential to generate particulate matter (PM) [166,167] and emit volatile organic compounds (VOCs), including toluene, aldehydes, and ethylbenzene [167,168]. Even widely used thermoplastics like ABS and PLA have been found to release VOCs [169]. Ensuring safe printing requires the careful management of chemical reactions and the corresponding emissions throughout the manufacturing process. In-depth studies aimed at unraveling the mechanisms of PM and VOC formation across different printing methods will guide the development of more environmentally friendly 3D printers, sophisticated control strategies, and enhanced personal protection measures.
